# Closed Mitral Valvotomy Reenvision

**DOI:** 10.7759/cureus.27401

**Published:** 2022-07-28

**Authors:** Swati Pathak, Rajeshwar Yadav

**Affiliations:** 1 Cardiacvascular Thoracic Surgery, Institute Of Medical Sciences, Banaras Hindu University, Varanasi, IND; 2 Cardiovascular Thoracic Surgery, Institute Of Medical Sciences, Banaras Hindu University, Varanasi, IND

**Keywords:** mitral stenosis in pregnancy, mitral valve surgery, percutaneous balloon mitral valvotomy, mitral stenosis, closed mitral valvotomy

## Abstract

In developing countries, like the Indian subcontinent, population overload, malnutrition, poor socio-economic status of affected groups, and health care facilities affect the treatment outcome. Nowadays procedures such as percutaneous balloon mitral valvotomy (PBMV) and open heart mitral valve replacement are offered to patients with mitral stenosis. Whenever PBMV is unavailable due to financial constraints and open surgical management cannot be offered due to overburdened healthcare facilities, closed mitral valvotomy (CMV) provides an excellent choice for patients with favorable mitral valve pathology. Many centers do not practice CMV and thus this procedure is dying out. The young generation of surgeons are not been trained in CMV. The purpose of our study is to reenvision CMV and emphasize its vital role in mitral stenosis patient subsets like pregnant women and young adults.

We reviewed the literature for various valvotomy techniques done for mitral valve stenosis and restenosis. Immediate and late outcomes were compared between the patients receiving Percutaneous balloon mitral valvotomy and closed mitral valvotomy.

The immediate and late-term results are comparable for PBMV and CMV and no statistically significant difference exists. The post-PBMV Mitral valve area (MVA) ranged from 2.1 +/- 0.7 cm^2 to 2.3 +/-0.94 cm^2 and post CMV MVA ranged from 1.3+/-0.3 cm^2 to 2.2+/-0.85 cm^2. Complications developing in both techniques are also nearly similar. Operative mortality in CMV patients ranged from 1% to 4.2%, also observed in PBMV patients in various studies. Mitral Regurgitation occurred in both groups equally and ranged from 0.3% to 14%. Restenosis was observed in both groups in the range of 4% to 5%. High fetal loss of around 20% mortality was witnessed in pregnant mitral stenosis patients undergoing open heart surgery.

It's time to re-envision CMV since it is providing substantial outcomes and remitting the need for open-heart surgery at a very low cost in patients with mitral stenosis with a pliable valve.

## Introduction and background

“To have the greatest number of patients live the longest time, in the best possible condition “ by Ankeney (1967). Seventy-three percent of the global burden of rheumatic heart disease (RHD) alone prevails in India, Pakistan, China, Indonesia, and the Democratic Republic of the Congo. Disability-adjusted life years due to RHD is approximate >10 million. Age-standardized prevalence is estimated to be maximum in females of the reproductive age group [1]. The prevalence of heart disease in pregnancy in developing nations is 0.65% (mitral stenosis due to RHD being the commonest ) [2]. In regions of a high prevalence of RHD, juvenile Mitral Stenosis is also frequently encountered (age ~ 5 years old). Treatment of valvular cardiac lesions as suggested in the American College of Cardiology (ACC) /American Heart Association (AHA) and European Heart Journal (ESC) valvular heart disease guidelines focuses on the identification and grading of the severity of the disease and early intervention [3]. Even in asymptomatic patients with moderate lesions, early intervention is indicated [4]. Hazards of cardiopulmonary bypass, anticoagulation, and poor health care referral facilities warrant us to opt for palliative intervention with comparable short and long-term results.

CMV completely justifies the above notion given by Ankeney as it caters to the greatest number of patients without posing a risk of anticoagulation. Surgical intervention with convincing short and long-term results along with low cost and less strict compliance and monitoring is the choice [5]. As compared to developed nations, in developing countries, especially in the Indian subcontinent population overload, malnutrition, poor socio-economic status of affected groups, and health care facilities affect the outcome of treatment [6]. Moreover, CMV offers the advantage of tactile assessment of the valve. What eyes cannot appreciate in the echocardiographic images, fingers can feel [7]. Twenty-five times less cost is required as compared to the open-heart procedure [7]. Closed Mitral Valvotomy therefore essentially remains the modality of treating the Mitral stenosis of favorable morphology as it addresses the above challenges. Where percutaneous procedures are unavailable due to financial constraints and open surgical management cannot be offered due to overburdened and limited resources Closed Mitral Valvotomy provides an excellent choice with convincing immediate and late outcomes in favorable mitral valve pathology.

## Review

Mitral valve stenosis: pathophysiology

The term valve (Latin: valvae) means double folding doors. Mitral (Italian: mitre) as it resembles a bishop’s mitre. In Mitral stenosis disease intervention is offered usually when [8]:

Mitral valve area (MVA) : < 1.5 cm2

Gradient (mean diastolic gradient): > 5 mm Hg

Pulmonary artery systolic pressure: > 50 mm Hg

Pulmonary capillary wedge pressure: > 20 mm Hg

Pulmonary arteriolar resistance : > 3 units

According to the Gorlin formula (1948); hence twofold increase in flow across the mitral valve causes a fourfold increase in the gradient across the valve. In pregnancy and labor; tachycardia increases the venous return after the uterus empties and sometimes when beta-agonist therapy is advised during labor [9], this increase in preload causes a manyfold increase in the gradient due to even a small increase of flow as there is fixed obstruction at the valve. Refractory pulmonary edema may ensue not responding to medical management. CMV is not only life-saving here but also prevents high fetal loss (~20% fetal mortality) which is evident in open-heart surgery [10].

Historical highlights

CMV is performed via left anterolateral thoracotomy incision in the fifth or sixth intercostal space. The mitral valve is digitally approached from the left atrium and Tubb's dilator is inserted through the left ventricle. The exploring finger guides the opening of the dilator. Valvotomy is achieved when dilator blades are opened to a preset value starting from 2.5 cm to 4 cm. Blades are dilated against a leaflet and not a commissure. CMV was the first intervention to be devised for mitral stenosis. In 1923 Cutler inserted valvotome and performed CMV for the first time [11]. Soutter did digital dilatation of stenotic lesion. After three decades Bailey in 1949 popularised this technique [12]. Tubb’s dilator was introduced in 1957, and since then the percentage of restenosis cases has declined, as the better release of fused commissures is possible with Tubb’s dilator as compared to the finger fracture method [13]. In 1952 Brock for the first time performed CMV on a pregnant patient. Abortion was earlier offered to such patients [14]. 

In PBMV the balloon is passed from the femoral vein to the right atrium via the inferior vena cava. From the right atrium through the septal puncture balloon reaches the Left atrium. The balloon is then negotiated through the mitral valve into the left ventricle. The balloon is configured such that, first the distal balloon inflates which is pulled back and hitched to the valve and then the proximal balloon is inflated which opens the commissures and increases the valve area. Percutaneous Balloon mitral valvotomy became available in 1990, introduced by Japanese surgeon Inoue.

Discussion

In a randomized controlled trial, Arora et al. [15] reported comparable short and long-term outcomes in 2000 patients that they studied. They equally divided the patients receiving PBMV and CMV. An increase in the mitral valve area was nearly comparable in both groups. The post-PBMV mitral valve area (MVA) ranged from 2.1 +/- 0.7 cm^2 to 2.3 +/-0.94 cm^2 and post CMV MVA ranged from 1.3+/-0.3 cm^2 to 2.2+/-0.85 cm^2. Complications like mitral regurgitation and restenosis were also the same in both groups. Mitral regurgitation occurred in both groups equally and ranged from 0.3% to 14%. Restenosis was observed in both groups in the range of 4% to 5%. Rifaie et al. [16] in a prospective randomized study in which they followed 40 patients for 15 years; reported similar immediate and long-term benefits in PBMV and CMV patients. Immediate postoperative increase in MVA ranged from 2+/-0.05 cm^2 in PBMV patients and 2.1+/-0.05 cm^2 in CMV patients. The mean diastolic pressure gradient was in the range of 6+/-4 mm Hg in both groups. Regression in mitral regurgitation in long-term follow-up was observed in both groups. Operative mortality in CMV patients ranged from 1% to 4.2%, also observed in PBMV patients in various studies. In seven years study by Farhat et al., a lower success rate was documented which reflected a lower immediate success rate and hence increased residual stenosis rate [16]. This study also highlighted the importance of subvalvular disease on the impact on the outcome. In a study carried out on 654 patients by Commerford et al. [17] low operative mortality (2.97%) and a survival rate of 90% were reported. Patients were followed for a period of 12 years. Stanley John studied immediate and long-term outcomes on 3724 patients: long-term survivors were 86%, and late deaths occurred in 4.3 %. CMV for restenosis was also carried out in their study which involved 6.7% of cases [18].

Fraser et al. elaborated on CMV for restenosis and had excellent outcomes in 70.5% of cases. Operative mortality in 10.4% and late mortality in 23.8 % of patients after a second CMV [13]. In Ellis and Harken's (1964) study of 139 patients in which a second CMV for restenosis was performed; late results were comparable to results after the first CMV [13]. R.K.Suri et al reported no role of PBMV in restenosis cases where they opted for the second CMV [5]. The interval between the first and second CMV was around 9.4 years. Improvement was documented in 89.4 % of cases. In a retrospective cohort study by Radhakrishnan, they reported results of mitral valve replacement after CMV. Hospital stay, ICU stay, and need for ventilation was found comparable in post-CMV and non-CMV MVR [19]. Aggarwal found excellent results of CMV in pregnancy and labor and also emphasized its cost-effectiveness [20]. They observed that acute pulmonary edema resulted in maternal death and when medical therapy failed CMV offered a great breakthrough in refractory patients. In a study 41 CMV were performed in the third trimester of pregnancy. All patients improved and no maternal mortality was documented with overall fatal survival of 87.8%. In a study by Otto with a focus on women with valvular heart disease, early intervention even in asymptomatic young females expecting pregnancy in near future was recommended. This would result in the prevention of symptoms and decompression during pregnancy [21]. In research by Naidoo et al, they reported how open heart surgery in pregnancy increases the risk of CNS damage, bleeding, and teratogenic effects due to anticoagulation. Thus indicating the role of PBMV/CMV in such patients [9].

Table 1 compares the pre and post-procedure mitral valve area achieved after PBMV and CMV.

**Table 1:** Mitral valve area (MVA) pre and post-PBMV and CMV in various studies PBMV-Percutaneous Balloon Mitral Valvotomy, MVA-MItral Valve Area (cm^2), CMV-Closed Mitral Valvotomy, NS -not significant

STUDY	J.J PATEL et al [22]	R.Arora et al [13]	Farhat et al [23]	Osama Rifaie et al [16]	N. Aggarwal et al [20]
PBMV (pre op MVA)	0.8 +/- 0.3	0.85 +/- 0.26	0.9 +/- 0.2	-	-
PBMV (post op MVA)	2.1 +/- 0.7	2.3 +/-0.94	2.1 +/- 0.5	2 +/- 0.05	-
CMV (pre op MVA)	0.7 +/- 0.2	0.79 +/- 0.21	0.9 +/- 0.2	-	0.8 +/- 0.2
CMV (post op MVA)	1.3 +/- 0.3	2.2 +/- 0.85	1.6 +/- 0.3	2.1 +/-0.05	2.1 +/- 0.01
p	<0.001	NS	-	NS	-
Mitral regurgitation (MR)	1 in each	12 (PBMV ) 14 (CMV)	-	-	-

Table 2 compares the operative mortality among the studies post CMV.

**Table 2:** Comparison of operative mortality

Study	Number of patients	operative mortality	Duration of follow up
Fraser et al [13]	359	4.2%	17 years
Suri et al [5]	113	2.8%	10 years
Stanley John et al [18]	3724	1.5%	5 years
P.J.Commerford et al [17]	654	2.97%	12 years
R. Arora et al [15]	2000	1%	22+/-6 months

## Conclusions

Amongst the various treatment options for mitral stenosis, CMV stands as the procedure of choice when our resources are limited and overburdened. With a short learning curve and the requirement of a smaller and simpler infrastructure, CMV should be practised by young surgeons. Instead of forgetting this simple procedure, it's time to reenvision CMV as a simple intervention providing substantial outcomes and remitting the need for open-heart surgery at a very low cost in patients with mitral stenosis with a pliable valve.
